# Fission in a colonial marine invertebrate signifies unique life history strategies rather than being a demographic trait

**DOI:** 10.1038/s41598-022-18550-9

**Published:** 2022-09-06

**Authors:** Oshrat Ben-Hamo, Ido Izhaki, Rachel Ben-Shlomo, Baruch Rinkevich

**Affiliations:** 1 Department of Evolutionary and Environmental Biology, Faculty of Natural Sciences, University of Haifa, Mount Carmel, 3498838 Haifa, Israel; 2 National Institute of Oceanography, Tel Shikmona, P.O. Box 8030, 31080 Haifa, Israel; 3 Department of Biology and Environment, Faculty of Natural Sciences, University of Haifa –Oranim, 36006 Tivon, Israel

**Keywords:** Ecology, Evolution

## Abstract

Each of the few known life-history strategies (e.g., r/K and parity [semelparity and iteroparity]), is a composite stratagem, signified by co-evolved sets of trade-offs with stochastically distributed variations that do not form novel structured strategies. Tracking the demographic traits of 81 *Botryllus schlosseri* (a marine urochordate) colonies, from birth to death, we revealed three co-existing novel life-history strategies in this long-standing laboratory-bred population, all are bracketed through colonial fission (termed NF, FA and FB for no fission, fission after and fission before reaching maximal colony size, respectively) and derived from organisms maintained in a benign, highly invariable environment. This environment allows us to capture the strategists’ blueprints and their net performance through 13 traits, each branded by high within-strategy variation. Yet, six traits differed significantly among the strategies and, in two, the FB was notably different. These results frame fissions in colonial organisms not as demographic traits, but as pivotal agents for life-history strategies.

## Introduction

Fission reflects a post-embryonic agametic division of an organism into distinct fragments, or ramets, that survive and regenerate into morphologically and functionally discrete organisms^1–3^; it is an event that is triggered endogenously (genetically programmed) or exogenously (environmentally imposed). This biological developmental phenomenon is widely distributed in marine invertebrate taxa, in both colonial^1,3–5^ and unitary organisms^6–9^. Studies have shown that fission can increase the number of field-counted organisms as much as, or more than, sexual reproduction can do^10–12^, and that ramets remain living and increase in local abundance, all of which has led to the understanding of fission as a demographic attribute or trait, primarily for clonal life-history strategies^13,14^.

The fission of clonal organisms appears to be a conservative trait across a variety of taxa and a wide geographical range^15^. Positive and negative demographic parameters and consequences arising from fission events in clonal organisms were described by metrics like reproductive outputs, colony growth, colony size, ramet fusion, survival rate, longevity, partial mortality and genet/ramet’s geometric-size constraints^1,4,12,16–19^; all portrayed fission as a pivotal developmental trait in the ecology and life history of many sessile invertebrates and plants^20^. In particular cases, the fission of colonies is posited as an effective ‘still be around’ approach or as a tool for enduring surviving in new colonization spaces^11^, an efficient way of offsetting the death of whole colonies^4^, or as a means to capture demographic advantages in the face of a wide range of environmental disturbances^19^, by permitting genets to escape local selective factors and poor-quality microhabitats^21^.

As in other sessile clonal organisms, colonial tunicates express a wide range of fission phenomena^22–27^, where additional small colonies are being more or less continuously generated in multiple ways^22,24^. Moreover, fission in tunicates has been linked to various demographic parameters, such as increased growth rates^22^, the long-term success of genets^24^, seasonality and alternating sexual/asexual reproduction^28^ and the influence of neighbors^29^.

Within the subphylum Tunicata, colonies of *Botryllus schlosseri*, a pan-European (now ecologically efficient cosmopolitan^30,31^) species, undergoes fission under both field and relaxed laboratory settings^23,28^, suggesting an endogenous trigger. Whole-genet size-regulating mechanisms in *B. schlosseri*, including fission, fusion and partial mortality, under natural and laboratory settings, further add abstractions to our understanding of the possible evolutionary trajectories that shaped fission events in this species^32^ or in other colonial species. The above traits dramatically alter a wide range of demographic parameters (e.g., the traditionally conjoined colony size and age), as well as affect resilience to environmental disturbances; yet, it is not experimentally established if fission is a solely demographic property, if it is a response to ecological drivers or whether it concurrently reinforces life-history attributes. Following fission events in 81 *B. schlosseri* colonies, which were maintained from birth to death in a controlled environment (laboratory cultures), we elucidated three novel and distinct fission-linked life-history strategies, each embodying disparate demographic properties.

## Materials and methods

### Animals

**Figure 1:**
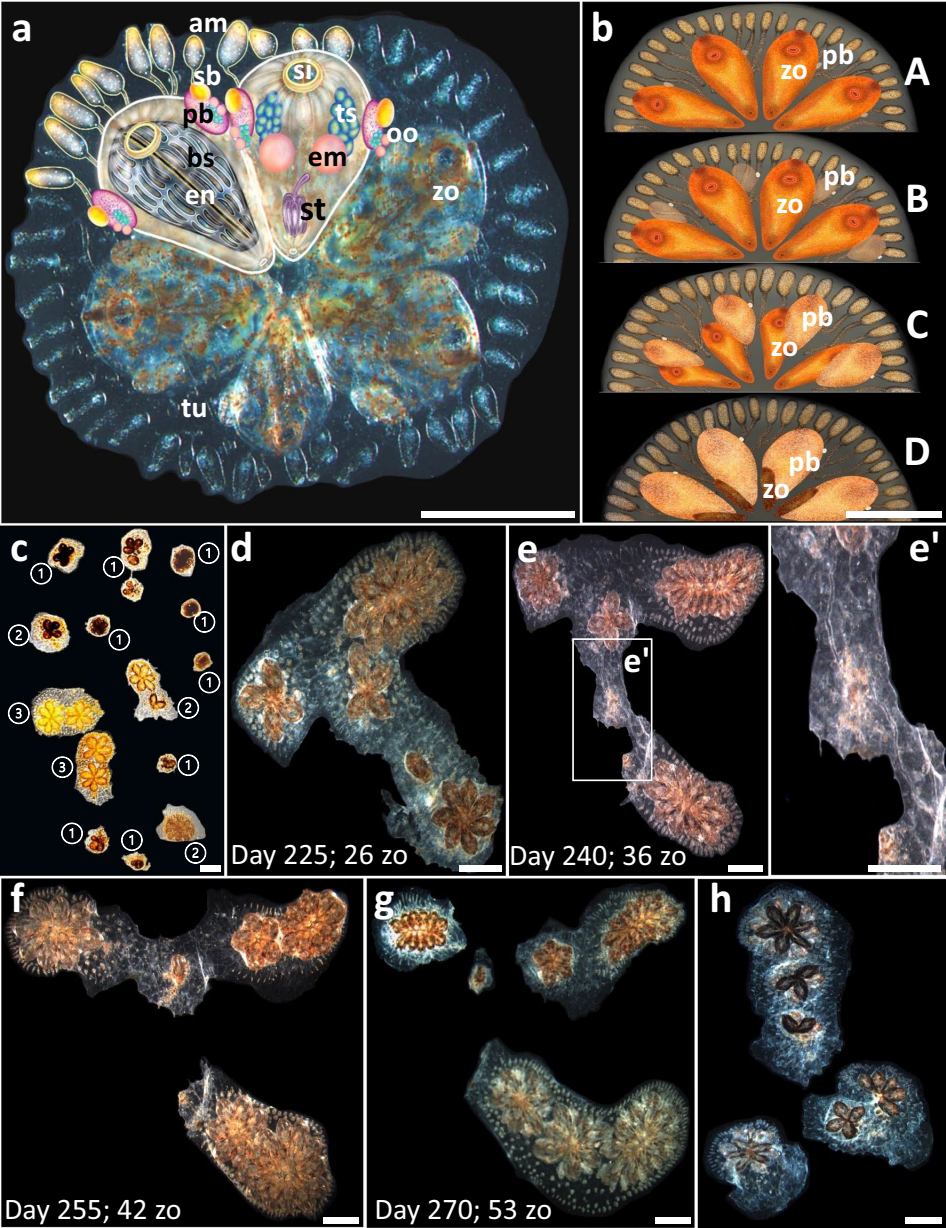
Blastogenesis and fission in *Botryllus schlosseri* colonies. (**a**) A general morphology of *Botryllus schlosseri*. The colonial entity is composed of zooids, clustered here in a single flower-like shape named “system”, containing six zooids (two zooids are schematically drawn, depicting internal organs). Three asexually derived generations are seen in the colony, the functioning zooids, the primary buds (pb), and the secondary buds (sb). Male gonads and female gonads are located in the zooids and buds, while embryos are found only in zooids. bs = branchial sac, em = embryo, en = endostyle, oo = oocyte, pb = primary bud, sb = secondary bud, si = siphon, ts = testes, tu = tunic, zo = zooid. (**b**) A colony is developed through blastogenesis, repeated cycles of life and death, each including four stages (A to D; *sensu*^62,63^). Each colony consists of three consecutive generations of modules at different developmental stages. Every blastogenic cycle lasts about 1 week at 20 °C and concludes in the death and absorption of the oldest generation, the zooids, while the younger generations, the buds and budlets, are developing. (**c**) A single *Botryllus* colony that underwent several fission events and is currently divided into 14 ramets. Ramets exhibit different CV scores (encircled numbers, see Suppl. Figure 1). The mean CV score of the colony (the sum of all ramets, the genet) is 2, taking into consideration the number of zooids in each ramet (**d**–**g**). Colony FB19 undergoing two fission events over four sequential observations. Age and genet size are mentioned in the lower-left part of each figure (zo = number of zooids). (**d**) Day 225: the colony can be seen as a single entity (**e**, e′). Day 240: the lower extension of the colony is retreat-growing (*sensu*^39^) from the upper part of the colony and both are still connected by a single blood vessel. (**f**) Day 255: the colony is split into two colonial ramets. (**g**) Day 270: a second fission event occurs in the upper ramet, resulting in three ramets. (**h**) A fissioned colony may demonstrate discrete blastogenic stages in the separate ramets, hypothetically enabling it to self-breed. The upper ramet is in blastogenesis stage C, the left ramet is in early blastogenic stage D and the right ramet is in blastogenic stage A. Scale bars = 1 mm.

We used laboratory bred *B. schlosseri* colonies (Fig. 1a), the offspring of colonies collected from marinas in California, USA, that established a vital stock of colonies for the research. The colonies were bred and maintained at 20 °C, under a 12/12-h light–dark regimen, inside 16 L seawater-filled plastic tanks in the facilities of the National Institute of Oceanography in Haifa, Israel, as described^33^. The colony's morphology and blastogenesis are detailed elsewhere^34,35^ (Fig. 1b). Oozooids were moved from their birthplaces, each onto a separate, tagged glass slide^36^. Hundreds of colonies were raised from the oozooid stage over a period of up to 3 years. From 1-month old, each colony was routinely observed (15 ± 5 days intervals) using a Nikon stereo microscope SMZ 1000. The observations included the number of zooids/buds, colonial fission, colonial vigorousness scores and reproductive statuses. New offspring settling on the glass substrates were immediately removed to avoid mixing them with the documented animals.

### Colonial vigorousness (CV) scores

CV scores are semi-quantitative metrics that summarize the morphological statuses of three major colonial compartments: the zooids/buds, the peripheral ampullae and the tunic, either in the whole colony or after fission, with the average taken of all ramets, that is the genet (Fig. 1c). The CV score for each genet ranged on a scale between 1 and 3 (low to high; Suppl. Figure 1), and in many observations (62%), the statuses of the major colonial compartments shared the same scale level. Following fission events, each CV score continued to reflect the overall genet’s score, taking into consideration the different ramet sizes, whereby each ramet’s score was multiplied by its zooid number, following which an entire genet's average CV score was calculated, providing more weight to larger ramets.

### Reproductive statuses (RS) scores

In developing *B. schlosseri* colonies, male gonads appear first, and two to four blastogenic cycles later, they were followed by the development of female gonads. Mature colonies may go through short or long periods of sexual sterility or may develop male gonads only^37,38^. Further, reproductive statuses (hermaphroditism, male-only, sexual sterility) may vary between different colonies under the same environmental conditions^37^. For the RS score, sexual sterility was graded as 0, male-only as 1 and hermaphroditism as 2. RS scores did not consider the average number of female/male gonads per zooid/bud. Observations were taken once every 15 days, such that the onset of reproductive activity in colonies could only be missed by a single blastogenic cycle (duration: 1 week at 20 °C). Following fission, an average RS score was calculated for the whole genet. In addition, we documented qualitatively the reproductive efforts’ outbreaks (RO). While we did not count the number of gonads/embryos as part of the observations, unusually high and fast changes in the number of ova in buds or embryos in zooids were observed and documented. Along the observations, we recorded extreme events where high numbers of embryos and/or ova per zooid were documented and these events were noted as “reproductive effort’s outbreak” to highlight qualitatively these episodes.

To delineate either a single or repeated sexual-reproduction cycle in colonies to reveal repeated male-only and hermaphroditic states, we defined each reproductive cycle as from the male-only state to the end of the subsequent hermaphroditic state, each termed as a RS segment (RSS).

### Colony fission

A fission event is characterized by a gradual splitting process, developing in the tunic matrix between colonial systems, following which the colonial entities, reaching a minimum size of two systems, were physically split into two or more disconnected ramets. Colony fission^37^ (Fig. 1d–h) may take days to weeks from onset to completion, and was determined to have begun with the first observation in which subclones of colonies were not connected by a single blood vessel. The number of zooids following fissions events revealed the sum of zooids in all ramets for any specific colony (genet).

### Statistical analyses

All analyses used SPSS software. A one-way ANOVA followed by a Bonferroni Multiple Sample Test were employed on the following characteristics: life span, age at the onset of male/female gonads, total zooid numbers, age at the peak of the colony's size, maximum colony size and RSS length (days). A one-sample t-test was used to determine the age at the first fission; a Mann–Whitney U test on the number of fissions throughout life; the non-parametric Kruskal–Wallis one-way ANOVA test employed on the number of RSS segments and observations with male-only/hermaphroditic states; Chi Square tests on the outcomes of fission events in the two-fissioned life histories; and discriminant analyses to predict the clusters of the three life histories.; Pearson tests determined correlations between life span and the number of all RSSs and between life span and the number of fissions; and Repeated Measures ANOVA was used on the gross size of colonies before and after the first fission.

## Results

### General

We followed 81 colonies growing on glass substrates from birth to death for up to 3 years (average life span was 290 ± 157 days, Table 1; Supp. Tables 1, 2, 3) under relaxed laboratory conditions. The onset of reproduction (male gonads) appeared within the first 2 months of age (55 ± 20 days; n = 65; Table 1) and oocytes first appeared about a month later (80 ± 51 days; n = 75; Table 1).

**Table 1:** Characteristics of the three life history strategies: NF—colonies that have not undergone a fission event throughout their entire lives; FA—colonies of which fission was observed after their maximum peak of zooids; FB—colonies that the fission was observed before their maximum peak.

Type	Life span (days)	Age at onset of male gonads (days)	Age at onset of female gonads (days)	RO (%)	Age at first fission (days)	Number of Fissions throughout life	Zooids no. along all observations	Age at peak of colony size (days)	Maximum colony size (no. of zooids)	RSS length (days)	Number of RSS segments	Observations (%) with male-only states in a RSS	Observations (%) with hermaphrodite states in a RSS
NF (n = 35)	189 ± 68^c^ (n = 35)	49 ± 14^b^ (n = 28)	70 ± 25^b^ (n = 32)	5^b^ (n = 35)	–	–	92 ± 41^c^ (n = 35)	99 ± 66^c^ (n = 35)	14 ± 5^c^ (n = 35)	75 ± 31^a^ (n = 30)	1.3 ± 0.6^c^ (n = 32)	34 ± 16^a^ (n = 30)	66 ± 16^a^ (n = 30)
FA (n = 23)	307 ± 146^b^ (n = 23)	53 ± 17^b^ (n = 17)	69 ± 19^b^ (n = 21)	13^ab^ (n = 23)	182 ± 79^a^ (n = 23)	1.3 ± 0.6^b^ (n = 23)	376 ± 203^b^ (n = 23)	133 ± 61^b^ (n = 23)	40 ± 25^b^ (n = 23)	86 ± 38^a^ (n = 42)	2.2 ± 1.5^b^ (n = 22)	33 ± 22^a^ (n = 31)	67 ± 22^a^ (n = 31)
FB (N = 23)	446 ± 164^a^ (n = 23)	68 ± 23^a^ (n = 20)	110 ± 81^a^ (n = 22)	32^a^ (n = 23)	158 ± 77^a^ (n = 23)	3.8 ± 2.3^a^ (n = 23)	812 ± 663^a^ (n = 23)	276 ± 105^a^ (n = 23)	62 ± 44^a^ (n = 23)	98 ± 53^a^ (n = 70)	3.2 ± 1.3^a^ (n = 23)	37 ± 20^a^ (n = 45)	63 ± 20^a^ (n = 45)
Overall averages	290 ± 157 (n = 81)	55 ± 20 (n = 65)	80 ± 51 (n = 75)	16 (n = 81)	168 ± 79* (n = 46)	2.6 ± 2.1* (n = 46)	373 ± 474 (n = 81)	159 ± 108 (n = 81)	35 ± 34 (n = 81)	89 ± 45 (n = 142)	2.1 ± 1.4 (n = 77)	35 ± 20 (n = 106)	65 ± 20 (n = 106)
Stat. test *p* value	A *p* < 0.001	A *p* = 0.001	A *p* = 0.001	K p = 0.021	T *p* = 0.963	M *p* < 0.001	A *p* < 0.001	A *p* < 0.001	A *p* < 0.001	A *p* = 0.289	K *p* < 0.001	K *p* = 0.616	K *p* = 0.784
F_*df*_/χ^2^ + *df*	F_*df*_ = 352	F_*df*_ = 82	F_*df*_ = 82	χ^2^ = 7.7 *df* = 2	F_*df*_ = 0.002_44_	F_*df*_ = 272	F_*df*_ = 972	F_*df*_ = 332	F_*df*_ = 612	F_*df*_ = 1.211	χ^2^ = 26 *df* = 2	χ^2^ = 0.969 *df* = 2	χ^2^ = 0.784 *df* = 2

RSS—Reproductive Status (RS) Segment. RO—Reproductive status outbreaks. χ^2^ = Chi square. A = One-way Anova. K = Kruskal–Wallis. T = One-sample T-test. M = Mann–Whitney U. Numbers of colonies are within parentheses; all values are given as means ± SD; superscript letters (a–c) mark statistically different groups (One-way ANOVA followed by Bonferroni post-hoc Test, *p* < 0.05 or Kruskal–Wallis Test followed by Mann Whitney U-tests); * = FA and FB averages only.

During their life spans, more than half of the colonies (n = 46; 57%) went through at least one fission event, including genotypes that showed repeated cycles of fast growth, degeneration (sensu^23,39^), rejuvenation (sensu^40^) and colony fissions. In the other 35 colonies, no single fission event was recorded throughout their life span. In the wake of the fission events, we defined three distinct life-history categories: (i) NF: colonies that did not experience a fission event throughout their entire lives; (ii) Fission type A (FA): colonial fission developed after the colony reached its peak number of zooids/observation; (iii) Fission type B (FB): colonial fission developed before the colony reached its peak number of zooids/observation.

### Fission as a demographic trait

The first signs of fission were witnessed when the typical colonial pattern of packed systems was interrupted, and each new developing colonial system in subsequent blastogenic cycles was spaced in the tunic matrix, consistently dispersed from neighbor systems (Fig. 1d–e′). Simultaneously, the semitransparent tunic turned opaque, developing imperfections in the fission areas (Fig. 1e).

Each colonial-fission process was completed within several days/weeks from its onset through a gradual tunic deterioration, occurring simultaneously with the narrowing of connecting vasculature and the reduction of blood-cell circulation, followed by the decay of the tunic and connecting blood vessels, until the actual disconnection between parting ramets (Fig. 1f–g). Within colonies that underwent fission, the first fission occurred at the age of 168 ± 79 days (n = 46) in colonies experiencing on average 2.6 ± 2.1 fission events throughout their life, and up to nine times per genotype (Table 1).

### Fission as a life-history strategy

**Figure 2:**
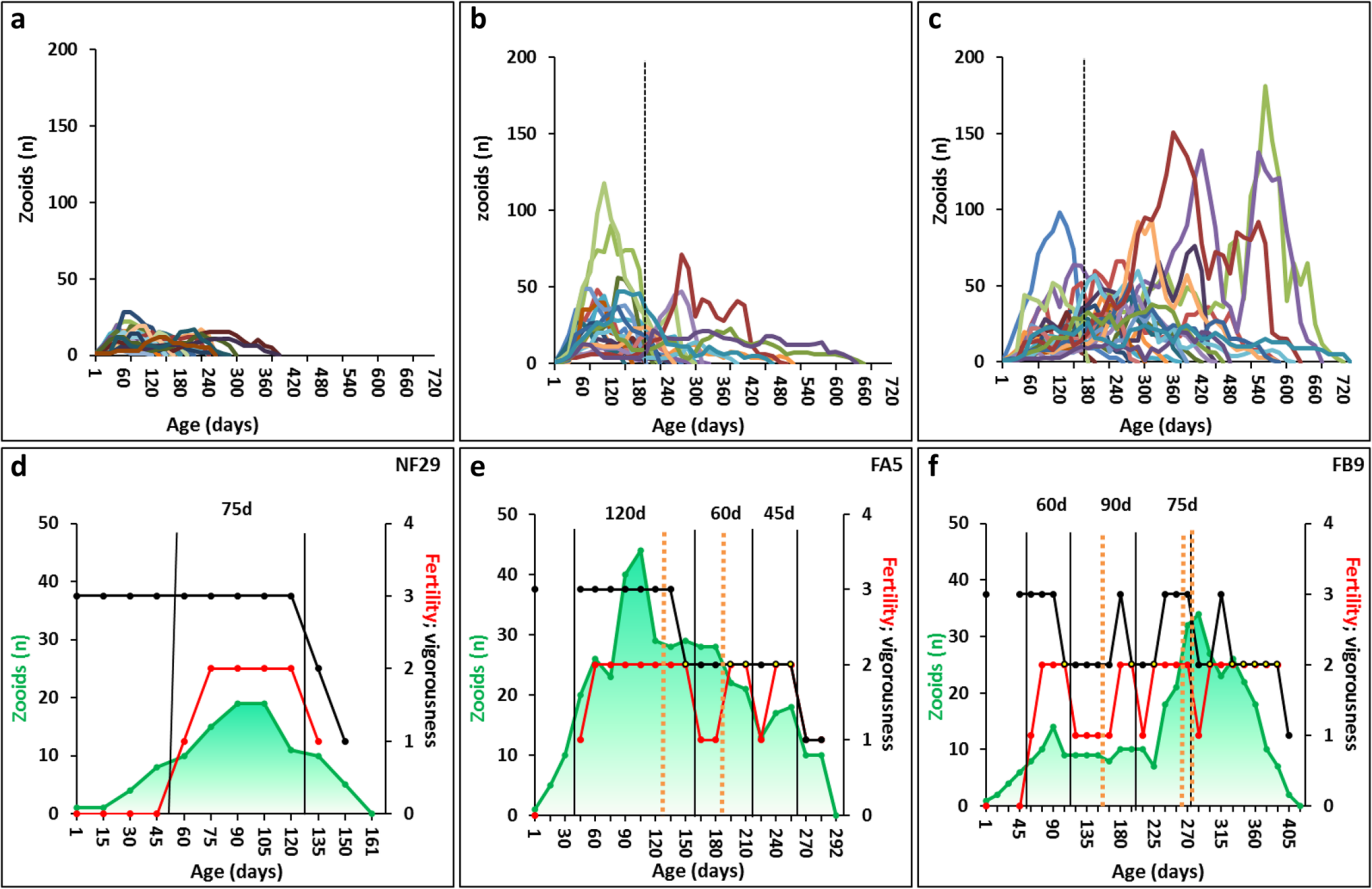
Life-history traits of the three strategies (**a**–**c**). Number of zooids in up to 17 data-collection sessions during the life spans of each of the 81 *Botryllus* colonies studied. The colonies are grouped into the three life-history strategies: (**a**) NF; (**b**) FA; (**c**) FB. The dashed vertical lines in (**b**) and (**c**) depict average ages for the first fission. (**d**–**f**) A typified representative of each of the three life-history strategies. (**d**) Colony NF29 as an example of the non-fission (NF) strategy. (**e**) Colony FA5 as an example for fission after zooids reach peak size (FA). (**f**) Colony FB9 as an example of fission before zooids reach peak size (FB). Life-history traits presented: the numbers of zooids (green curve), RS scores (red curve) and CV scores (black curve). Vertical black lines outline RS segments (RSS), with the length (days) marked for each RS segment. Vertical dotted orange lines depict fission events.

Life expectancy, the total number of zooids during a life span, the average age of reaching maximal colony size and the maximal colony size differed significantly between the three life-history strategies (n = 35 for NF, n = 23 for FA, and n = 23 for FB colonies; Table 1, Fig. 2a–c, Suppl. Tables 1, 2, 3). NF colonies are marked by the shortest life span (189 ± 68 days), by the lowest total number of zooids during a life span (92 ± 41), by the youngest age for reaching the maximal colony size (99 ± 66 days), and by the smallest maximal colony size (14 ± 5 zooids), while FB colonies typified the longest/highest values (life span: 446 ± 164 days; total number of zooids: 812 ± 663; age at peak of colony size 276 ± 105 days; maximum colony size: 62 ± 44 zooids; Table 1). Further, FB and FA (fissioned strategies) were significantly disparate from each other in the number of fissions over a life span (3.8 ± 2.3 vs. 1.3 ± 0.6; FB vs. FA, respectively) but not in the age of the first fission (Table 1, Fig. 2a–c, Suppl. Tables 2, 3). NF and FA significantly differed from FB at the age of the onset of male and female gonads (Table 1). Reproductive outbreaks (RO) were also significantly disparate between NF, FA and FB colonies (Kruskal–Wallis; *p* = 0.021; Table 1). A Mann–Whitney U Post hoc revealed a significant difference between NF and FB (*p* = 0.008).

A typical NF pattern shows a single peak in the number of zooids around midlife, followed by a gradual, constant decline, to a minimal colonial size before death, as well as a single RSS, composed of a male-only phase, followed by a 2-month-long hermaphroditic status, spanning the time of the colony's peak size (Fig. 2a,d). The fissioned FA and FB strategies are epitomized by repeated RSSs of high and low CV scores that repeatedly decline at the end of each RSS and raise at the beginning of a new segment, when colonies re-juvenilize, as is manifested by an upsurge in the number of zooids (data not shown). RSSs contained several male-only observations that were inserted between hermaphroditic states (FA: Fig. 2b,e; FB: Fig. 2c,f). Sexual-sterility phases (excluding the pre-reproductive stage of young colonies) were rare, recorded in 2 NF, 1 FA and 4 FB colonies, all at advanced ages, and which were accompanied by a significant zooid reduction. Further, fission was commonly followed by the loss of developmental synchronicity between ramets of the same genet (e.g., Fig. 1h).

**Figure 3:**
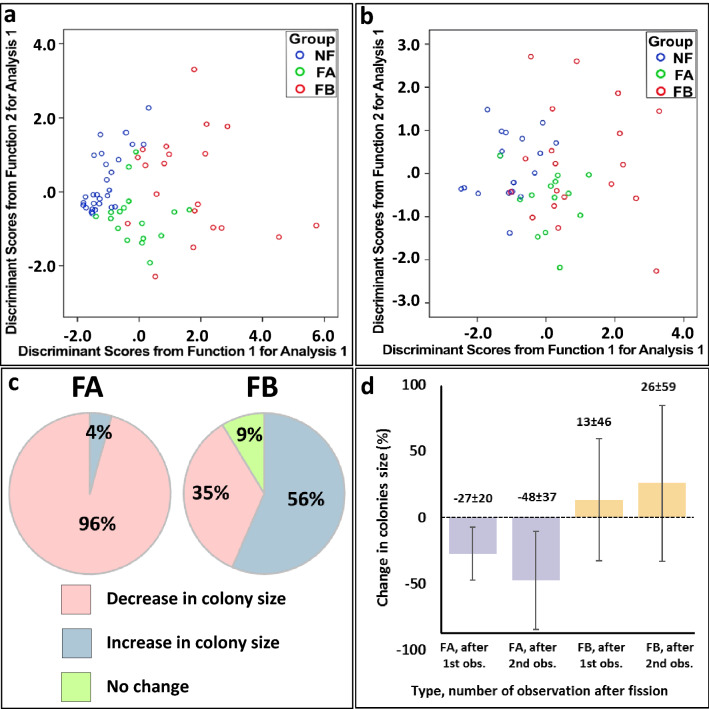
(**a**,**b**) Discriminant analyses outputs cluster the already assigned three life-history strategies of *Botryllus* colonies according to: (**a**) age at maximal colony size, life span, maximal size (number of zooids) and ages at the onset of male and female gonads (days). Colony numbers: NF = 28, FA = 17, FB = 20. (**b**) number of segments throughout the life, length of segments (days), number of times in a segment in which the male-only state is observed and number of times in a segment in which the hermaphroditic state is observed. Colony numbers: NF = 18, FA = 13, FB = 19. (**c**) Impact of the first fission on sizes of colonies in FA or FB colonies. The differences in the frequencies of the outcomes (increase/decrease) between the types are significant (chi-test, chi square = 16.8, *df* = 1, *p* < 0.001). (**d**) Mean (± SD) change in size of FA and FB colonies (percent; y axis) in the two observations following the first fission event. The changes are relative to the number of zooids in the observation that preceded the fission event.

A discriminant analysis of 65 colonies with complete data, which comprised the variables length of life span, age at the peak colony size, maximum size of the colony, ages of the onset of male gonads and of female gonads (Suppl. Table 1, 2, 3), further clustered, such that 81.5% of the colonies shared their assessed strategy (27 of the 28 presumed NF colonies, 12 of the 17 FA colonies and 14 of the 20 FB colonies; Wilks’ Lambda *p* < 0.001; Fig. 3a).

### Characterization of the RS segments (RSSs)

A single RSS (delineated by vertical black lines in Fig. 2d–f) was found in 74% of the NF colonies, 50% in the FA colonies and 9% in the FB colonies. The average RSS length (89 ± 45 days for 142 RS segments in 62 colonies) did not significantly differ between the three life-history strategies, even though the mean RSS in FB was 31% longer than in NF (Table 1, Suppl. Table 4).

By contrast, the number of RSSs significantly differed between the three life-history strategies (Table 1, Suppl. Table 1). The Pearson correlation coefficient (r) for life span compared to the number of all RSSs was 0.8 (n = 77, *p* < 0.001), demonstrating a strong positive relationship between the two variables. Yet, examining proportions of male-only and hermaphroditic observations within a segment (Table 1; 57 colonies with 106 RSSs) revealed 35 ± 20% and 65 ± 20%, respectively, with no significant difference between the three life-history strategies (Table 1), suggesting that reproductive statuses within segments are not a variable trait when considering the colonial strategic life span.

A discriminant analysis (Fig. 3b) of the four RSS variables (segment length [days], number of segments/genet, number of observations for male-only state/segment and for hermaphroditic state/segment; Table 1, Suppl. Tables 1, 2, 3) for all colonies with complete data (n = 50, 18 NF, 13 FA and 19 FB colonies) revealed three significantly disparate strategies (Fig. 3b; *p* < 0.001). The analysis clustered 72% of the colonies with their suggested life-history pattern (15/18, 10/13 and 11/19 of NF, FA and FB colonies, respectively).

### Fissions shape astogeny

A Pearson correlation between life span and the number of fissions in FB colonies revealed a correlation coefficient (r) of 0.68 in FB (*p* < 0.001) compared to 0.33 for FA (*p* = 0.1). Yet, the time of the first fission (a baseline fission, as additional fissions did not develop in all ramets) in all fissioned types (n = 46) was 168 ± 79 days (Table 1), with no significant differences between the FA and FB strategies (Table 1), suggesting an inherent fission trait in astogeny. Focusing on the first fission event, in 96% of FA colonies, fission was followed by an immediate (the first observation following fission) decrease in colonial size, whereas 62% of fissions in FB colonies resulted in an immediate increase in colonial size (Fig. 3c; chi-test, chi square = 16.8, *df* = 1, *p* < 0.001), an outcome further supported by analyses performed on the first and second fission events (Fig. 3d; F = 4.4; *df* = 1.8, 12.8; *p* = 0.019). While total sizes of FA colonies declined after fission (before: 235 ± 155 zooids; after: 99 ± 88 zooids), in FB colonies, total sizes increased (before: 146 ± 78 zooids; after: 616 ± 626 zooids). A Repeated Measures ANOVA test on the changes in the means, revealed no significant difference between the two time-points in the two fission types (F = 0.369, *df* = 1, *p* = 0.547).

## Discussion

All life-history strategies have been marked by co-evolved sets of trade-offs, which are the constrained lineages between traits, revealing an ‘optimization’ of trade-offs for growth, survival and reproduction^41–43^. Clearly, an optimal life-history strategy may be different for each species, depending on its genetic background, past and present environment, and other biological and environmental interactions and constraints. Yet, these life-history strategies illustrate the flexibility of within-species life histories, which allow organisms to endure various conditions and survive strong stochastic-disturbance events^44–46^. Flexible life-history characteristics among individuals of a specific taxon can further influence the magnitude of demographic stochastics, maximizing the extent to which offspring of parents with distinct life-history strategies are differentially staged at the population level, and further conferring diverse fitness constraints^46–48^.

The interplay of survival, life span, growth rate and reproductive status define life-history strategies across taxa. Each such life-history strategy is a composite stratagem and banks on the coordination of several co-functionating traits. While the literature attests for a high diversity of demographic properties and traits within populations and large amounts of phenotypic variation and trade-offs among individuals, with regards to size, survival, onset of reproduction and total reproductive outputs^41,45,48–50^, the number of life-history strategies remains minimal, as the different variations and constraints revealed by different taxa distribute stochasticity, without forming novel structured strategies along a continuum (such as r and K strategies, or parity, semelparity and iteroparity^51,52^.

The reiteration of modules and the ability of colonial organisms to create independent ramets provide colonies with the proficiency to develop, like weeds, bushes, or trees, bodily structures that solitary animals cannot^53^. While individuals of unitary organisms appear to hold different positions along the r and K strategy continuum of life-history strategies^43,46,47,50,54^, with the employment of fission, fusion and partial mortality, colonial organisms may "deceive" with regards to the correlations between age/size/reproduction^1,3,4,55^ and thus rebuff the accepted notion of structured life-history traits for large vs. small phenotypes in colonial organisms.

**Figure 4:**
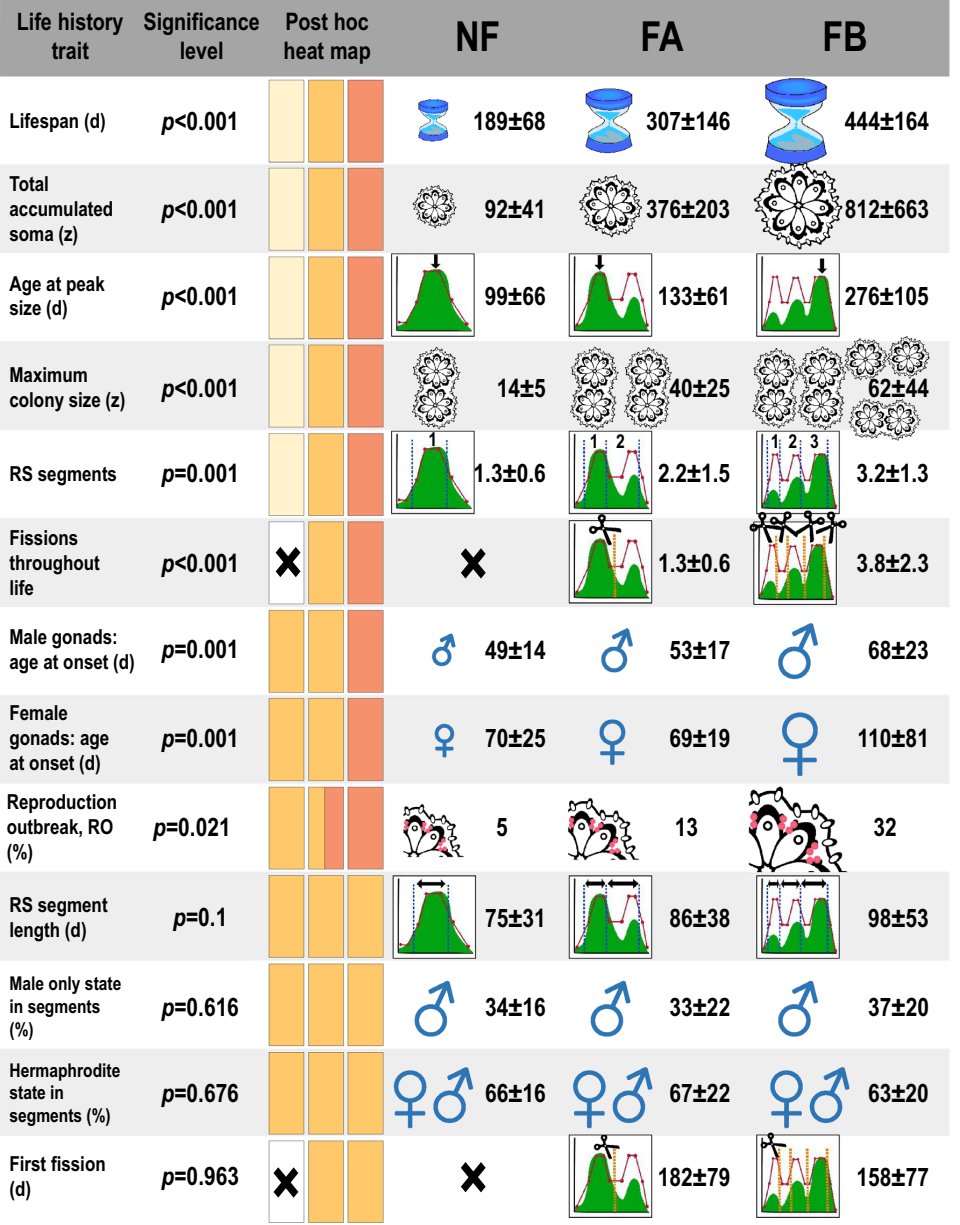
The major traits’ epitomes that describe the three life histories (NF, FA and FB) as expressed by *B. schlosseri* colonies maintained under relaxed environmental settings. Traits are organized according to *p* values, from those portraying three statistically significant groups to traits that do not differ between the life histories. Significant post-hoc test results are visualized by the heat map (from left to right: NF, FA, FB) where different colors indicate significantly different groups. Where possible, self-explanatory graphical icons depict the major outcomes, with size differences revealing the statistical differences. d = days; z = zooids; RS = Reproductive statuses; RO = Reproduction outbreak; NF = colonies that did not fission throughout their lives; FA = colonies that fissioned after reaching peak numbers of zooids; FB = colonies that fissioned before reaching peak numbers of zooids.

Three alternative life-history strategies (NF, FA, FB; Fig. 4) which reflect genetically based ecological consequences, co-exist in a long-term laboratory-bred *B. schlosseri* population. All are categorized through the phenomenon of colonial fission (no fission, fission after/before reaching maximal colony size) and derived from organisms maintained under benign, further assumed to be highly invariable, environmental (laboratory) conditions. NF, FA and FB strategies thus underlie the core expression of the traits’ phenotypes. Moreover, the benign environment allows for the capture of the genetic blueprints of these strategies and their net performance (e.g., fission has been developed without any recognized biological or environmental driver, contrasting with existing literature; see^56^; Herrera-Cubilla et al.,^57^; Bingham et al.,^58^). The *Botryllus* NF type is short-lived and reaches maximum size and sexual maturity at an early age. The NF type also has the smallest accumulated soma size and the fewest RS segments over its life. By contrast, the FA type is a phenotype with a medium-length life span that reaches maximum size at a mid-age and sexual maturity at an early age. The FA type also has an intermediate accumulated soma size and a middling number of RS segments over their lives. Finally, the FB type is a long-lived phenotype that reaches maximum size and sexual maturity later in life. The FB type also has the highest accumulated soma size and the most RS segments over their lives.

It has been advocated that functional ecological traits could be related to any species’ demography^59^, an argument primarily evidenced in field-oriented studies (e.g.^60^). Rather than defying demographic trade-offs, this study presents basic associations between fission and divergent life-history traits in *B. schlosseri* in a relaxed environment, configuring the framework for each of the three coexisting, starkly different (Fig. 4), yet variable, life-history strategies. Thirteen traits were followed, branded by high within-strategy variation, yet six traits (life span, total accumulated soma, age at peak size, maximum colony size, RS segments and number of fission events) differed significantly between the three strategies. In two traits (ages of onset of male/female gonads), the FB was notably different, and only four traits (RSS length, male-only state in segments, hermaphroditic state in segments and age at first fission) were not significantly different from each other (Fig. 4). The expression of high variability associated with any trait, as exemplified by the 81 genets that were followed here from birth to death, reveals a continuous range for each trait’s metric (broached as phenotypic plasticity), together with a clear distinction between the three life-history strategies.

The main conclusion of the present study is that fission in colonial organisms should not be solely understood within the framework of a demographic trait *per se*^10,14,56–58^ but should preferably be considered as a trait along a continuum (*sensu*^43,46^), reflecting a major attribute of a life-history strategy. Observing the expression of fission under relaxed (benign) environmental conditions allowed us to identify colonial fission with the framework of distinct life-history strategies, in concert with a suite of other continuously varying traits (Fig. 4). Even with the expressed high within-trait variability, altogether the results revealed three finely graded but distinct life-history strategies. These intra-specific life-history strategies in *B. schlosseri* and their landscapes’ variations, with aligned ratcheting fitness (a topic not studied here), are likely reflected in the successful invasiveness of this species, which has become cosmopolitan in temperate zones^30,61^.

## Supplementary Information

Supplementary Information.
