# Supplementary material for: Fission in a colonial marine invertebrate signifies unique life history strategies rather than being a demographic trait
Source: Sci Rep. 2022 Sep 6;12:15117. doi: 10.1038/s41598-022-18550-9 (PMC9448763; doi:10.1038/s41598-022-18550-9)
Supplement: Supplementary file 1 — Supplementary Information. [file 41598_2022_18550_MOESM1_ESM.pdf]

Supplementary figure and table for the article: “Fission in a colonial marine invertebrate signifies unique life history strategies rather than being a demographic trait”

Authors names: Oshrat Ben-Hamo, Ido Izhaki, Rachel Ben-Shlomo, Baruch Rinkevich

Suppl. Figure 1. Detailed descriptions for the *B. schlosseri* colonial vigorousness (CV) scores, as revealed from biological statuses assigned to three major bodily compartments: the tunic, the peripheral ampullae and zooids/buds. Scale bars=1 mm

**Colonial compartment**	**CV score**		
	**1**	**2**	**3**
**Tunic**	Whitish and opaque shade, amorphous texture flattened or swollen, deteriorated and partly damaged, peeled-off substrates. No active ampullae.<br>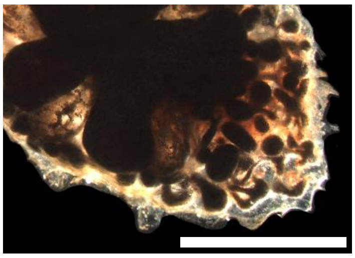	Cloudier, murky shade. May be swollen and/or partly damaged. Less active ampullae. Accumulation of cells.<br>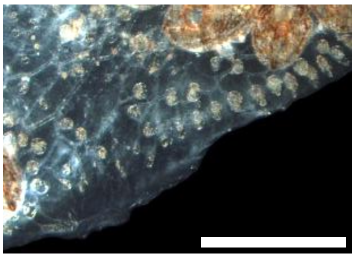	Transparent and Intact. No tissue vestiges. Intersperse with active ampullae.<br>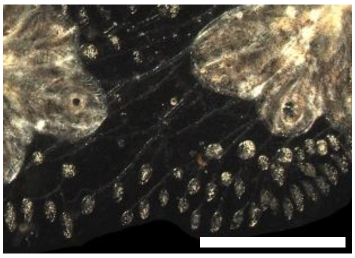
**Peripheral ampullae**	Much fewer, abnormal size: rounded, and shrunk. Sluggish cell movement. Cell leakage to the tunic.<br>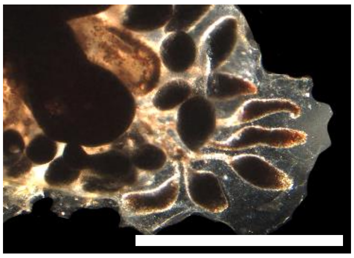	Less active movement. Abnormal sizes either larger or smaller. Circulating cells reveal more intense colors, pigment cells increase. Less crowded, partly remoted from tunic edges<br>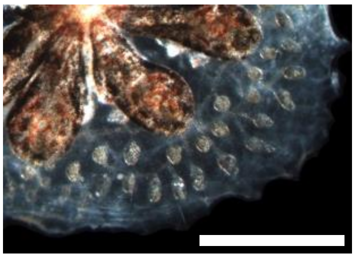	Actively moving, swollen, elongated and crowded, covering most of the tunic periphery at its edge. Fast cell movements. Most circulating cells are transparent or with blunt colors.<br>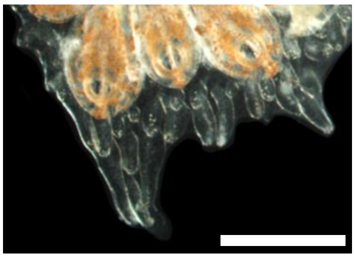
**Zooids and buds**	Shrunk sizes and abnormal shapes. Many zooids are dispersed in the tunic. May not present in the same blastogenic phase. Highly pigmented. 0-1 buds/zooid.<br>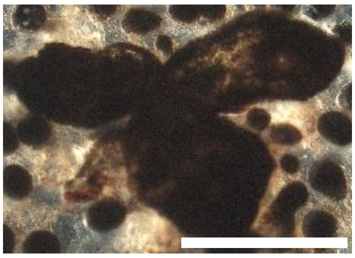	Colony pattern formation is partly impaired. Not all zooids may present at the same blastogenic phase. Slightly to medium pigmented.1-2 buds/zooid<br>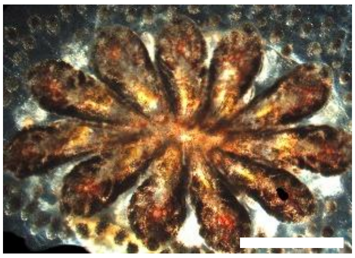	Fully assembled in compact flower-like systems. Developmental stages of all zooids/buds are synchronized, within the same blastogenic phase. Transparent shades to slightly pigmented. Actively moving in the tunic. The cell islands around the endostyle are clearly seen. Two or more buds/zooid.<br>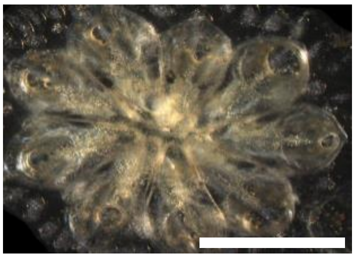

Suppl. Table 1. Metrics for the 35 NF colonies, including: lifespan (days); Maximum size (no. of zooids); Age at peak of colony size (days); Age at onset of male gonads (days); Age at onset of female gonads (days); Sum of all zooids ever counted; Number of RS segments. NI = no information is available.

Name	Lifespan (d)	Maximum size (no. of zooids)	Age at peak of colony size (d)	Age at onset of male gonads (d)	Age at onset of female gonads (d)	Sum of all zooids ever counted	No. of RS segments
NF1	110	16	60	27	34	65	1
NF2	180	10	49	35	40	59	2
NF3	133	22	63	35	49	122	1
NF4	110	20	40	NI	35	80	2
NF5	133	16	40	30	45	90	1
NF6	167	16	76	NI	NI	75	NI
NF7	140	28	76	70	85	151	1
NF8	170	13	45	45	59	93	1
NF9	131	19	75	45	59	103	NI
NF10	176	13	120	65	85	95	1
NF11	176	15	137	46	137	82	NI
NF12	162	10	46	NI	46	64	1
NF13	145	8	63	63	63	51	1
NF14	145	8	102	70	70	50	1
NF15	162	13	86	65	65	70	1
NF16	191	9	70	31	46	84	2
NF17	243	15	86	NI	NI	113	1
NF18	243	17	220	NI	107	140	1
NF19	200	6	27	27	70	40	1
NF20	235	15	120	NI	NI	143	1
NF21	175	8	120	30	70	71	1
NF22	160	12	28	50	70	77	1
NF23	170	8	120	55	103	65	1
NF24	131	8	43	NI	45	41	1
NF25	120	4	30	43	43	16	1
NF26	190	15	133	41	70	90	1
NF27	147	12	42	42	42	65	1
NF28	147	15	57	57	70	80	1
NF29	161	19	100	60	70	103	1
NF30	260	14	85	56	70	89	3
NF31	380	15	240	46	70	218	2
NF32	297	14	230	30	55	156	2
NF33	391	11	285	55	108	157	3
NF34	280	17	205	60	95	142	2
NF35	244	12	148	60	100	95	2
mean	188.7	13.5	99.1	49.0	69.5	92.4	1.34
STD	68.2	4.8	66.4	13.7	24.5	41.0	0.6
N	35	35	35	28	32	35	32

Suppl. Table 2. Metrics for the 23 FA colonies, including: lifespan (days); Maximum size (no. of zooids); Age at peak of colony size (days); Age at onset of male gonads (days); Age at onset of female gonads (days); Sum of all zooids ever counted; Number of RS segments; Age at first fission (days). NI = no information is available.

Name	Lifespan (d)	Maximum size (no. of zooids)	Age at peak of colony size (d)	Age at onset of male gonads (d)	Age at onset of female gonads (d)	Sum of all zooids ever counted	No. of RS segments	Age at first fission (d)
FA1	259	37	89	30	40	296	1	134
FA2	157	48	91	30	49	257	1	118
FA3	217	90	122	48	59	688	1	179
FA4	323	20	77	NI	65	121	3	121
FA5	292	44	110	70	70	422	3	130
FA6	502	22	90	45	80	258	4	136
FA7	247	27	93	28	41	208	2	120
FA8	192	17	163	NI	70	122	1	176
FA9	204	56	137	70	70	337	1	163
FA10	243	16	85	85	101	155	1	106
FA11	147	27	41	41	70	224	1	133
FA12	159	40	109	55	55	317	1	115
FA13	185	49	71	71	85	474	1	146
FA14	247	21	205	70	86	157	3	235
FA15	291	118	106	NI	NI	871	2	160
FA16	307	47	271	NI	85	356	3	300
FA17	386	22	115	NI	NI	280	NI	130
FA18	244	24	190	47	47	297	1	231
FA19	218	37	134	59	70	380	1	190
FA20	487	71	270	48	65	629	3	355
FA21	648	26	115	NI	110	542	6	160
FA22	636	22	214	43	55	606	4	400
FA23	458	47	150	56	85	645	5	238
Mean	306.5	40.3	132.5	52.7	69.4	375.7	2.2	181.8
STD	145.7	25.0	60.9	16.5	18.6	202.7	1.5	79.0
n	23	23	23	17	21	23	22	23

Suppl. Table 3. Metrics for the 23 FB colonies, including: lifespan (days); Maximum size (no. of zooids); Age at peak of colony size (days); Age at onset of male gonads (days); Age at onset of female gonads (days); Sum of all zooids ever counted; Number of RS segments; Age at first fission (days). NI = no information is available.

Name	Lifespan (d)	Maximum size (no. of zooids)	Age at peak of colony size (d)	Age at onset of male gonads (d)	Age at onset of female gonads (d)	Sum of all zooids ever counted	No. of RS segments	Age at first fission (d)
FB1	254	98	120	41	55	669	2	99
FB2	580	66	255	70	147	1065	3	167
FB3	714	181	550	80	100	2129	6	175
FB4	661	139	416	100	130	2574	4	146
FB5	372	25	220	45	65	287	2	136
FB6	347	43	205	61	70	402	1	45
FB7	264	29	200	65	100	329	2	157
FB8	191	33	137	NI	86	203	2	121
FB9	418	34	286	59	70	389	3	159
FB10	444	76	405	70	130	1036	3	110
FB11	391	43	269	50	70	529	4	200
FB12	290	39	220	NI	72	307	2	138
FB13	392	34	311	NI	NI	451	3	108
FB14	271	34	235	55	80	369	3	145
FB15	173	52	107	59	70	472	1	96
FB16	455	43	285	42	107	518	3	27
FB17	604	68	285	70	70	818	5	157
FB18	579	93	318	59	70	1110	2	219
FB19	579	52	265	59	70	840	4	245
FB20	623	151	358	70	445	2339	4	232
FB21	458	38	310	138	165	737	5	179
FB22	471	20	415	100	153	403	4	415
FB23	726	26	185	71	85	704	5	159
Mean	446.0	61.6	276.4	68.2	109.5	812.2	3.2	158.0
STD	164.2	43.5	104.8	22.9	81.4	663	1.3	76.9
n	23	23	23	20	22	23	23	23

Suppl. Table 4. Lengths of RS segments (days) in colonies of the three life histories (NF, FA and FB).

	RS segment Length (d)
NF1	45
NF2	60
NF2	75
NF3	60
NF4	NI
NF4	30
NF5	NI
NF6	NI
NF7	NI
NF8	NI
NF9	NI
NF10	60
NF11	NI
NF12	NI
NF13	NI
NF14	NI
NF15	60
NF16	45
NF16	105
NF17	NI
NF18	NI
NF19	135
NF20	NI
NF21	90
NF22	NI
NF23	NI
NF24	45
NF25	30
NF26	90
NF27	NI
NF28	75
NF29	75
NF30	60
NF30	45
NF30	45
NF31	105
NF31	135
NF32	90
NF32	120
NF33	90
NF33	45
NF33	135
NF34	90
NF34	90
NF35	75
NF35	45
MEAN	75.0
STD	30.8
n	30

	RS segment Length (d)
FA1	195
FA2	NI
FA3	150
FA4	75
FA4	30
FA4	45
FA5	120
FA5	60
FA5	45
FA6	75
FA6	60
FA6	45
FA6	105
FA7	135
FA7	NI
FA8	120
FA9	105
FA10	105
FA11	90
FA12	75
FA13	90
FA14	75
FA14	30
FA14	45
FA15	NI
FA15	NI
FA16	105
FA16	45
FA16	105
FA17	NI
FA18	NI
FA19	90
FA20	150
FA20	150
FA20	60
FA21	90
FA21	45
FA21	60
FA21	135
FA21	75
FA21	NI
FA22	45
FA22	60
FA22	90
FA22	105
FA22	NI
FA23	120
FA23	45
FA23	NI
FA23	45
FA23	105
MEAN	85.7
STD	38.4
n	42

	RS segment Length (d)
FB1	105
FB1	NI
FB2	45
FB2	75
FB2	90
FB2	225
FB3	60
FB3	90
FB3	240
FB3	45
FB4	105
FB4	45
FB4	225
FB4	120
FB5	90
FB5	90
FB6	150
FB7	105
FB7	60
FB8	60
FB8	45
FB9	60
FB9	90
FB9	75
FB10	105
FB10	30
FB10	150
FB11	105
FB11	30
FB11	30
FB11	75
FB12	75
FB12	120
FB13	NI
FB13	90
FB13	120
FB14	105
FB14	45
FB14	60
FB15	75
FB16	90
FB16	165
FB16	75
FB17	90
FB17	45
FB17	105
FB17	75
FB17	180
FB18	150
FB18	210
FB19	165
FB19	105
FB19	90
FB19	105
FB20	180
FB20	210
FB20	60
FB20	75
FB21	45
FB21	45
FB21	60
FB21	45
FB21	45
FB22	75
FB22	60
FB22	105
FB22	105
FB23	105
FB23	60

FB23	240
FB23	120
FB23	60
MEAN	97.9
STD	52.8
n	70
